# Investigation on Performance of Hydraulically Expanded Joint of Titanium–Steel Clad Tubesheet

**DOI:** 10.3390/ma16031106

**Published:** 2023-01-27

**Authors:** Jia Li, Juan Li, Yuyan Zhang, Changyu Zhou

**Affiliations:** 1 School of Mechanical and Electronic Engineering, Nanjing Forestry University, Nanjing 210037, China; 2 School of Mechanical and Power Engineering, Nanjing Tech University, Nanjing 211816, China

**Keywords:** TA2-Q345R clad tubesheet, expanded joint, residual contact pressure, groove width

## Abstract

The performance of a hydraulically expanded joint between tubesheet and titanium tube was analyzed using a finite element numerical calculation. The connection strength of Q345R tubesheet and TA2-Q345R clad tubesheet was studied using a tight expansion method. The results proved that the residual contact pressure and pullout force of the tight expansion joint of TA2-Q345R clad tubesheet were greater than those of the Q345R tubesheet. However, the residual contact pressure of the expanded joint without a groove for the TA2-Q345R tubesheet and the pullout force failed to meet the requirement of connection strength. Hence, the groove was employed on the contact surface. The influences of groove position and groove width on the connection strength of the expanded joint with grooves in tubesheet hole were studied. The results show that the residual contact pressure of the clad tubesheet of grooving in the cladding layer was higher than that of grooving in the base layer. The effect of the position of groove in the cladding layer and base layer on the residual contact pressure could be neglected. A wider groove led to a higher residual contact pressure, which increased significantly when the groove width was 4 mm.

## 1. Introduction

Titanium is an alloy with low density, high strength, and strong corrosion resistance, which is widely used in the chemical industry, marine ships, aviation, medical instruments, automotive industry, livelihood supplies, and other fields [1,2]. Because of its good economic and applicability, titanium-steel clad tubeplate structures are applied in titanium shells and tube heat exchangers.

The joint between the tube and tubesheet is the key part of the shell and tube heat exchanger, which is also prone to failure. Expansion is an important type of connection between the tube and tubesheet, which can be divided into hydraulic expansion, mechanical expansion, and explosion expansion. Among them, hydraulic expansion is a uniform flexible expansion method. The expansion pressure is easy to control and does not damage the inner wall surface of the expansion; thus, the joint is of good quality.

How to improve the sealing performance and tensile resistance of hydraulic expanded joints has always been the focus of research. Therefore, many research achievements have been made on the influence of material properties [3,4], machining accuracy [5,6], geometric parameters [7,8], manufacturing process [9,10], and other factors on the performance of joints. In order to improve the connection strength and sealing performance of the tube-to-tubesheet joints, tubesheet holes are grooved [11,12]. Material properties, especially plasticity, play a crucial role in obtaining accurate results close to true values [7]. Before finite element analysis, it is important to determine material properties such as yield stress [13]. Previous studies have shown that strain hardening is an important factor influencing the accuracy of joint numerical analysis results [14,15]. Previous studies mainly focused on the combination of a carbon steel tube and low-alloy steel tube sheet, or the combination of a titanium tube and single-material tube sheet. For the combination of titanium tube and titanium–steel clad tubesheet, we can find reports on the stress analysis [16,17], creep of titanium [18], etc. However, there are few reports on the impact of the titanium–steel clad tubesheet cladding layer and grooving parameters on the performance of expanded joints. Jawad et al. [7] used experimental methods to study the expanded joint of titanium–steel clad tubesheet; the best result was obtained when the groove width was equal to $1.56\sqrt{r_{o}t}$, where r_o_ and t are the outside radius and thickness of the tube, respectively. The residual contact stress of titanium–steel clad tubesheet with a groove in the base layer was calculated by numerical simulation [19]. The above studies did not discuss the influence of groove form on titanium–steel clad tubesheet joint performance. By means of an experiment, Ma Qiulin [20] found that TA2 had the characteristic of elastic hysteresis after loading, and its residual contact pressure decreased with the increase in time after the completion of titanium tube expansion. Subsequently, the experimental results of the same research team [21] showed that, under 260 MPa hydraulic expansion pressure, after 96 h of unloading the expansion pressure, the pullout force of the joint decreased by 60% and 23%, corresponding to the expanded joint without grooves in the tubesheet hole and with two grooves in the tubesheet hole, respectively. Therefore, the residual contact pressure at the moment of expansion completion should not be used as the only basis for analyzing the performance of expanded joints, and the negative effect caused by elastic hysteresis should also be considered. Therefore, in this paper, the hydraulic expanded tube joint of a titanium–steel clad tubesheet is taken as the research object. Through numerical simulation, the sealing performance and connection strength of the tight expansion (joint without groove) are studied; furthermore, the joint with grooves is analyzed. The influence of the expansion method and structural parameters, including groove form, groove width, and groove location in the base, is investigated, which provides a basis for the optimization of the expansion structure of the clad tubesheet.

## 2. Finite Element Model Analysis

### 2.1. Material Performance

**Figure 1:**
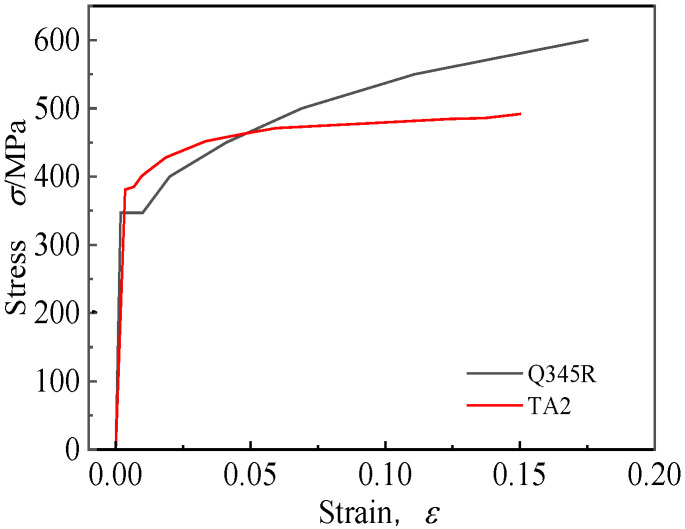
Tensile property curves of materials.

The tube was made of commercial pure titanium TA2, and the titanium–steel clad tubesheet material was TA2-Q345R, in which the base material was Q345R and the cladding material was TA2. The physical properties of the two materials are shown in Table 1. The true stress and strain of material were simulated using multilinear isotropic reinforcement material, and their mechanical property curves are shown in Figure 1.

### 2.2. Finite Element Model

**Figure 2:**
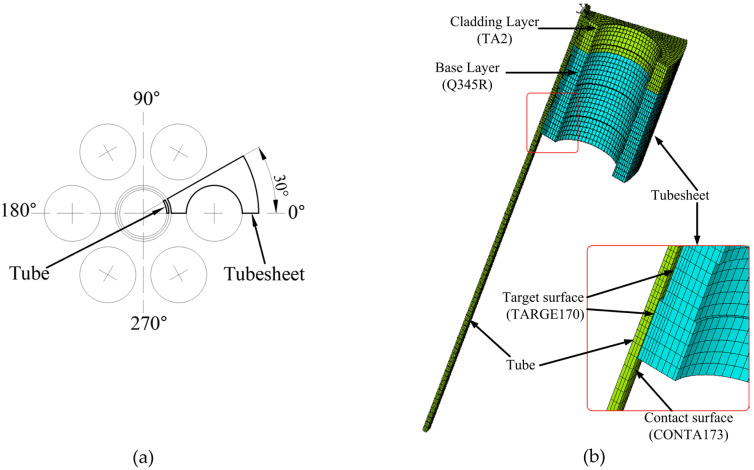
Finite element analysis: (**a**) analysis scope; (**b**) finite element mesh model.

The tubes were arranged in regular triangles. Due to the periodicity of pipe drainage, a seven-hole model was adopted, and 1/12 of the circle, i.e., a 30° area, was taken as the research object, as shown in Figure 2a. The size of the tube was φ25 mm × 1.5 mm, and the length of the tube was 180 mm. The diameter of the tubesheet hole was 25.3 mm, the distance between the tube centers was 32 mm, the outer diameter of the tubesheet was 480 mm, and the total thickness of the tubesheet was 50 mm with a 38 mm base layer and a 12 mm cladding layer, conforming to the recommended scope of the standard [22].

Because the overall structure of all joints was similar, only the finite element model of the joint with grooved clad tubesheet holes is shown in this manuscript. The SOLID185 element was used to establish the finite element model of the expanded joint, as shown in Figure 2b. The mesh was finely divided near the expansion surface. The combination of the outer wall of the titanium tube and the inner wall of the tubesheet hole was a nonlinear contact problem. The outer wall of the titanium tube was the contact surface, and the CONTA173 contact surface element was selected. The inner wall of the tubesheet hole was the target surface, and the TARGE170 target surface element was selected. The contact algorithm adopted the Augmented Lagrange method, and the appropriate solution value was guaranteed by controlling the contact stiffness FKN and the maximum allowable penetration value FTOLN [23,24].

During expansion, expansion pressure was applied on the inner surface of tube; the tube end was subjected to axial and circumferential symmetric constraints, whereas the outer cylindrical surface of the tubesheet was axially constrained, and the remaining surfaces were free.

After expansion, the expansion pressure on the tube was removed. Under the pulling condition, the axial displacement constraint of the tube was removed, axial (Z-direction) displacement was applied, and the other boundary conditions remained unchanged. During calculation, an automatic step and large deformation were chosen, and a linear search to stabilize the calculation and the complete Newton–Rapson method were used.

## 3. Tight Expansion Analysis

### 3.1. Theoretical Calculation Method

Yan Huigeng’s [25] theoretical calculation method of residual contact pressure based on the double-cylinder model is widely used for the calculation of residual contact pressure of the tight expansion, as shown in Equation (1).

(1) $$p_{c}^{*}=(1-2c)p_{i}-\frac{2}{\sqrt{3}}\sigma_{\mathrm{st}}\ln K_{t},$$

Here,

$$c=1/\{K_{t}^{2}(1-\mu_{t})+1+\mu_{t}+\frac{E_{t}(K_{t}^{2}-1)}{E_{s}(K_{s}^{2}-1)}[1-\mu_{s}+K_{s}^{2}(1+\mu_{s})]\},$$

where *σ*_st_ is the yield strength of the tube material (MPa), *K*_t_ = *r*_o_/*r*_i_ is the diameter ratio of the heat exchanger tube, *r_o_, r_i_* are respectively the inner and outer diameters of the heat exchanger tube, *K*_s_ = *R*_o_/*R*_i_ is the diameter ratio of the equivalent cylinder, *R_o_, R_i_* are respectively the inner and outer diameters of the equivalent cylinder, *μ*_t_, *μ*_s_ are the Poisson’s ratios of the tube and tubesheet material, respectively, and *E*_t_, *E*_s_ are respectively the elastic moduli of the tube and tubesheet material (MPa).

**Figure 3:**
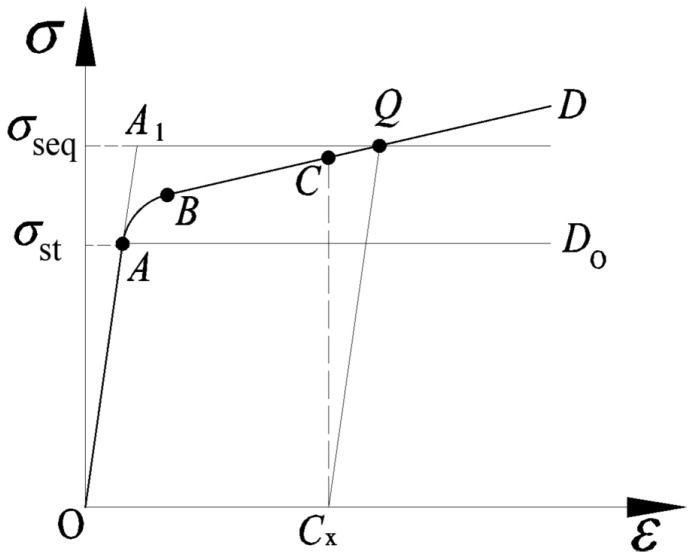
Schematic diagram for determining the equivalent yield strength.

However, Yan Huigeng’s theoretical calculation method assumes that the tube material is an ideal elastic–plastic material, and *σ*_st_ in Equation (1) is the yield strength of the tube material without considering the strain strengthening of the material; thus, the theoretically calculated value of *p*_c_^*^ is greater than the real value. Hao Junwen [13] proposed the concept of pipe equivalent yield strength *σ*_seq_ according to the research methods of predecessors [25], i.e., replacing *σ*_st_ in Equation (1) with *σ*_seq_, as shown in Figure 3.

As shown in Figure 3, the curve $\bar{OABCD}$ represents the true stress–strain curve of the titanium tube. The section $\bar{OA}$ is the elastic deformation stage, the section $\overset{⌢}{AB}$ is the partial plastic deformation stage, and the section $\bar{BD}$ is the full strain strengthening stage. Assuming that the tube contacts the tubesheet when the stress and strain develop to point *C*, and that the abscissa *C*_x_ = *g*/*r*_o_ corresponding to point *C* is the strain value of the outer wall of the tube, *g* is the clearance between the outer wall of the tube and the inner wall of the hole. By making a straight line with a slope of *E*_t_ through the *C*_x_ point, the straight line intersects the curve at point *Q*. Then, the ordinate *Q*_y_ corresponding to point *Q* is the equivalent yield strength of the tube material *σ*_seq_. According to the tensile curve of TA2 in Figure 1, *σ*_seq_ = 415 MPa, which is 35 MPa different from the original value of 380 MPa.

### 3.2. Comparison of Simulation Results and Theoretical Calculation Results

**Figure 4:**
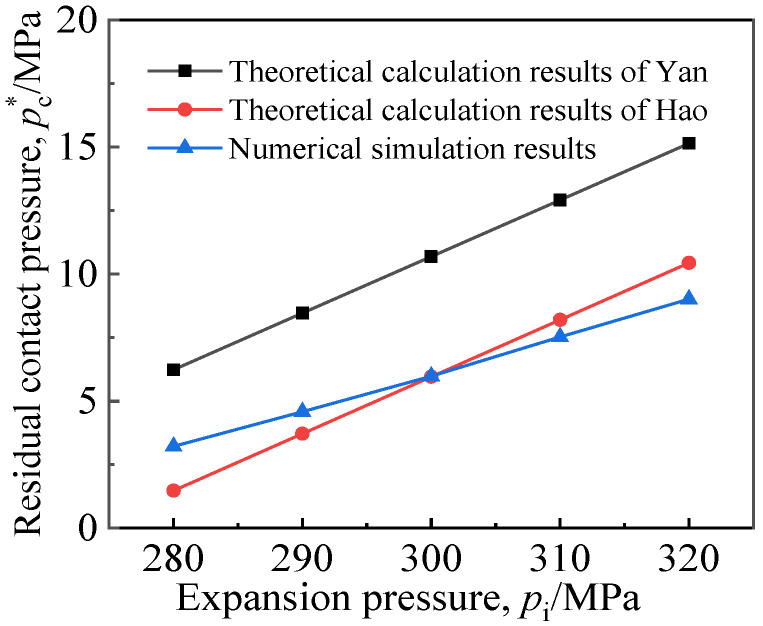
Comparison of calculation results [13,25].

The experiment and numerical simulation results show that the friction coefficient *f* is related to many factors such as the accuracy of the machined surface and the properties of materials. According to the experimental data of the expansion connection between TA2 tube and Q345R tubesheet in the literature [20], the friction coefficient *f* was set to 0.28. The *p*_c_^*^ with tube material TA2 and tubesheet material Q345R was simulated, and the results were respectively compared with the theoretical results of Yan [25] and Hao [13], as shown in Figure 4. It can be seen that the simulation value was much smaller than the value of Yan’s theoretical method, and it was mainly consistent with the result of Hao’s theoretical method, which is closer to the real situation. Therefore, the parameters of the simulation and the results were considered reasonable.

### 3.3. Performance Analysis of Tight Expansion Joint 

**Figure 5:**
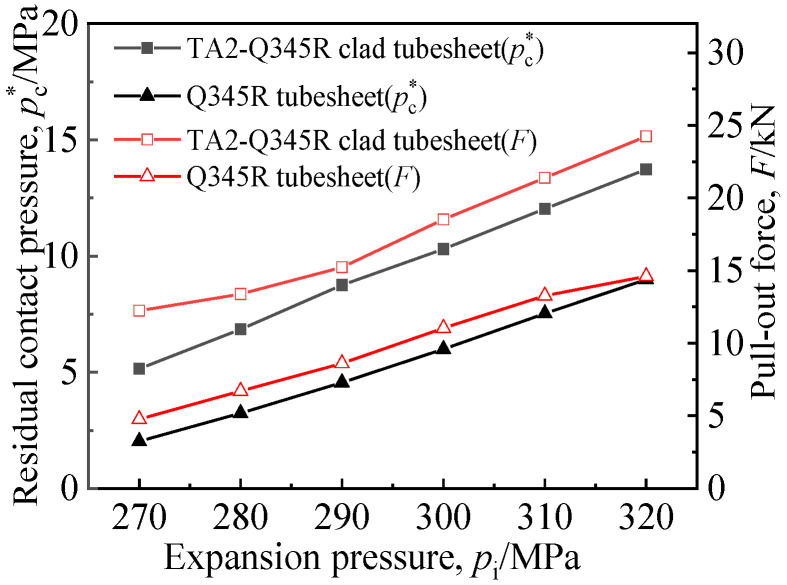
Tight expansion joints performance of two kinds of tubesheets.

In order to investigate the influence of the cladding surface on the sealing performance of the expansion joint, the expansion and pulling processes of the joints between the TA2 tube and Q345R tubesheet, and between the TA2 tube and TA2-Q345R clad tubesheet under different expansion pressures were simulated. The average *p*_c_^*^ on the contact surface and pullout force *F* were obtained, as shown in Figure 5.

It can be seen from Figure 5 that *p*_c_^*^ and *F* of expanded joints of different materials increased with the increase in expansion pressure, and *p*_c_^*^ and *F* of the TA2-Q345R clad tubesheet were both higher than those of the Q345R tubesheet under the same expansion pressure.

The pullout force provided by the expanded joint is expressed in Equation (2).

*F* > π*dl*[*q*]. (2)

According to GB/T151-2014 “Heat Exchanger” [26], the allowable pullout force [*q*] of the steel heat exchange tube of tight expansion is 2 MPa; thus, the pullout force should satisfy *F* > 7.23 kN according to Equation (2). For titanium expansion joints, the pullout force decreases with the time after the expansion due to the elastic hysteresis effect. A previous study [21] gave the relationship curve between the pullout force and time after the expansion of the titanium tube and steel tubesheet. Considering that the pullout force of the joint decreases by about 75% during the period from the completion of manufacturing until service, the corresponding pullout force of the titanium tube should be greater than 28.92 kN at the moment of completion of the expansion. As can be seen from Figure 5, the pullout force of the joint still failed to meet this requirement even when the expansion pressure exceeded 320 MPa.

**Figure 6:**
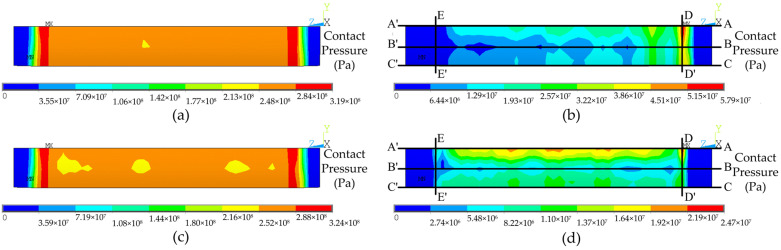
Contact pressure distribution on the contact surface of two kinds of tubesheets: (**a**) TA2-Q345R clad tubesheet after loading; (**b**) TA2-Q345R clad tubesheet after unloading; (**c**) Q345R tubesheet after loading; (**d**) Q345R tubesheet after unloading.

Figure 6 shows the contact pressure distribution on the contact surface of the TA2-Q345R clad tubesheet or Q345R tubesheet after loading and after unloading. According to Figure 6a,c, when the expansion pressure reached 320 MPa, there were two upper and lower sealing rings on the contact surface of the two tubesheet conditions, consistent with the simulation results in [4,27]. When the expansion pressure was unloaded, it can be seen from Figure 6b,d that there was a residual contact pressure ring on the contact surface of the tube side, but this was not obvious on the shell side. This is because the stiffness of the tube on the shell side was greater than that on the tube side; hence, it was not easy to expand and deform. It can also be found from Figure 6b,d that the residual contact pressure of the sealing ring of the TA2-Q345R clad tubesheet after unloading was greater than that of the Q345R tubesheet (57.9 MPa > 24.7 MPa) because the cladding layer of the clad tubesheet and the tube were the same material TA2. When the expansion pressure was removed, the tubesheet rebounded with the tube; thus, the contact pressure of the cladding part could maintain high values. Therefore, although the contact pressure of the base part decreased substantially because the elastic modulus of TA2 was less than that of Q345R, the average residual contact pressure in the whole contact range of the clad tubesheet was higher because the residual contact pressure of the cladding part was high, and the corresponding pullout force was also higher.

**Figure 7:**
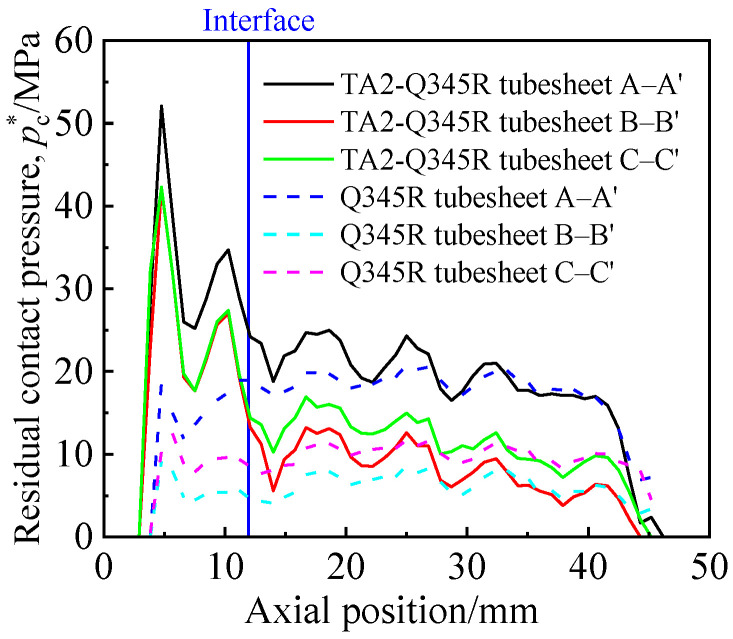
Axial distribution of residual contact pressure of the two kinds of tubesheets.

Three axial paths A–A′, B–B′, and C–C′ (in Figure 6) were taken at 0°, 15°, and 30° of the circular direction of the contact surface, and the residual contact pressure along the three paths was extracted as shown in Figure 7. It can be seen from Figure 7 that the highest contact pressure of the two tubeplates was at the same position in the axial direction (Z-direction). Compared with the Q345R tubesheet, the effective sealing range of the tube side of the clad tubesheet was closer to the expansion edge, the *p*_c_^*^ value at the sealing ring was much higher than that of the Q345R tubesheet, and the *p*_c_^*^ value of the middle and shell side of the two kinds of tubeplates was similar.

**Figure 8:**
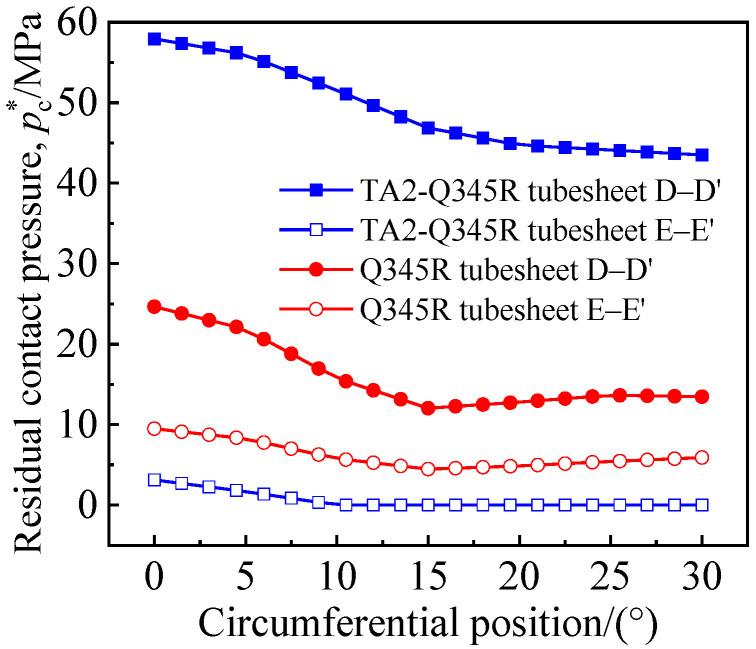
Circumferential distribution of residual contact pressure of Q345R tubesheet and TA2-Q345R clad tubesheet.

Figure 8 indicates the *p*_c_^*^ on the circumferential paths D–D′ (tube side) and E–E′ (shell side) on two sealing rings. The results show that the *p*_c_^*^ of the shell side of the two tubesheets was low, and the *p*_c_^*^ at some point on the TA2-Q345R clad tubesheet was zero, indicating that, with the rebound of the TA2 tube after unloading, the sealing ring at some positions on the contact surface of the shell side gradually disappeared. When the heat exchanger is in service, the shell side medium would leak along the contact surface and extend to the pipe side, which would damage the contact surface and cause hidden dangers to the safe operation of the equipment. Therefore, the method of ungrooved hole tight expansion was not suitable for the titanium–steel clad tubesheet, and it was necessary to groove the tubesheet hole for strength expansion to improve the joint connection strength. 

## 4. Expanded Joint with Grooved Clad Tubesheet Holes

**Figure 9:**
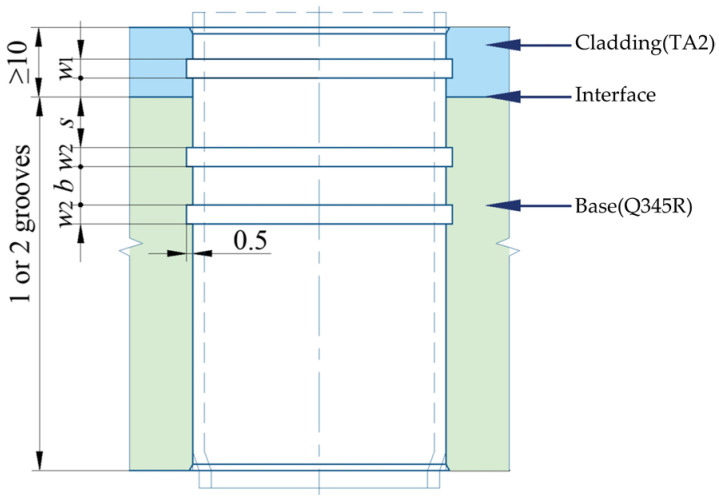
The structure and dimension of grooves.

**Figure 10:**
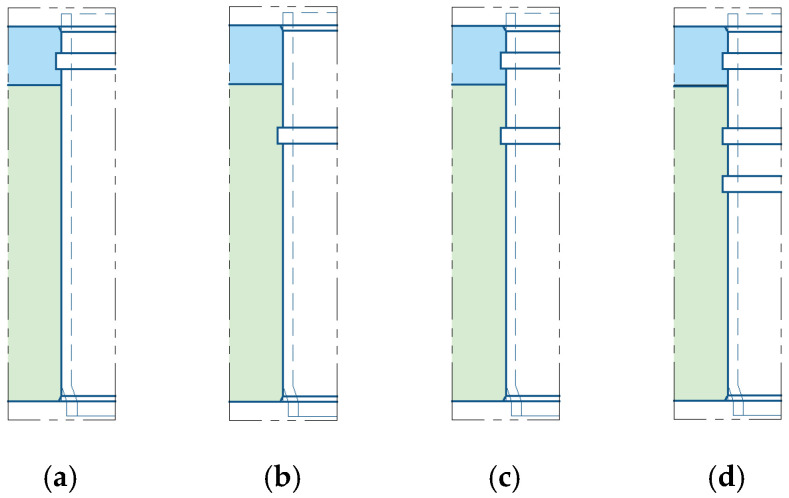
Four schemes for grooving: (**a**) one groove in the cladding layer only; (**b**) one groove in the base layer only; (**c**) one groove in the cladding layer and one groove in the base layer; (**d**) one groove in the cladding layer and two grooves in the base layer.

In the standard GB/T 151-2014, the structure and dimension of the circumferential grooves in clad tubesheet holes are as shown in Figure 9. In order to study the effect of groove form on residual contact pressure and pullout force, four groove forms were designed, as depicted in Figure 10. Figure 10a–d feature one groove in the cladding layer only, one groove in the base layer only, one groove in the cladding layer and one groove in the base layer, and one groove in the cladding layer and two grooves in the base layer, respectively. The effects of groove width *w*, groove distance *s*, and groove spacing *b* on the connection strength and sealing performance are also investigated. The groove dimensions are listed in Table 2. 

### 4.1. Effect of Groove Form

**Figure 11:**
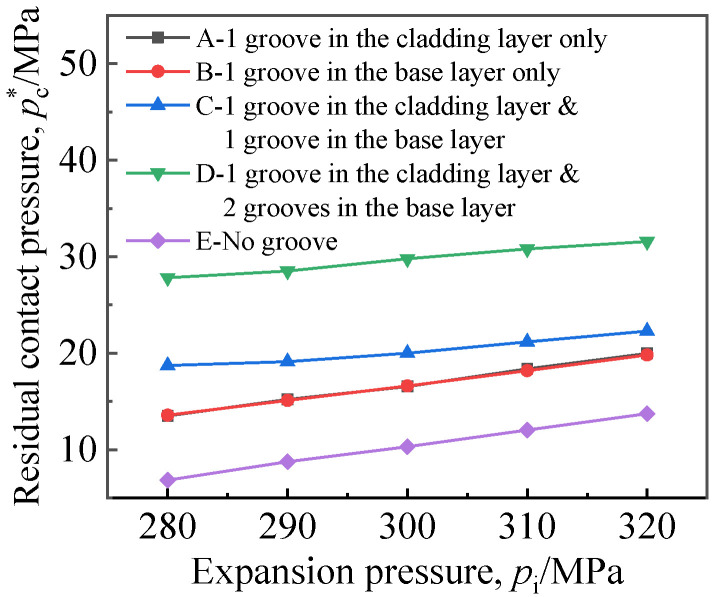
Effect of groove location on residual contact pressure.

**Figure 12:**
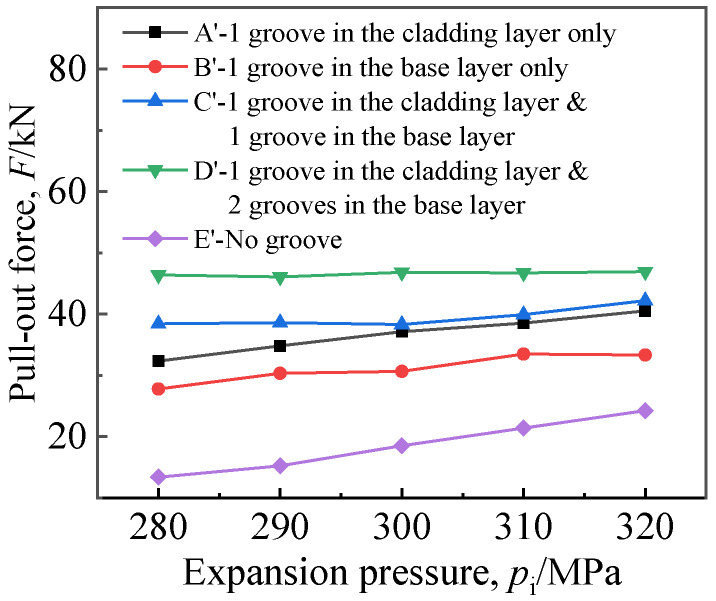
Effect of groove location on pullout force.

When a single groove was in the base layer or the cladding layer, the groove width *w*_1_ or *w*_2_ was 8 mm; when both the cladding layer and the base layer were grooved, the base layer groove width *w*_2_ was 8 mm, and the cladding layer groove width *w*_1_ was 6 mm. Figure 11 and Figure 12 illustrate the residual contact pressure and pullout force at the moment of the expansion pressure being unloaded.

Figure 11 shows that joints with grooved holes had a higher residual contact pressure *p*_c_^*^ than those without grooved holes, and *p*_c_^*^ was positively correlated with expansion pressure. Lines A and B coincided, indicating that grooving in the base layer or the cladding layer had little effect on the average residual contact pressure. When there was a groove in the cladding layer, the grooving in the base layer significantly improved *p*_c_^*^, and the double grooving in the base layer was best.

As shown in Figure 12, the pullout force *F* almost increased with *p*_i_, and *p*_i_ had a weak influence on *F* when the number of grooves was two or three. Under the same expansion pressure, the relationship of pullout force at different grooving locations was as follows: *F* for one groove in the cladding layer and two grooves in the base layer > *F* for one groove in the cladding layer and one groove in the base layer > *F* for one groove in the cladding layer only > *F* for one groove in the base layer only > *F* for no grooving. The pullout force of grooved joints was greater than 28.92 kN; therefore, when the strength expansion with a groove width of 8 mm was applied to titanium tubes, even when considering the decline in the pullout force caused by elastic hysteresis, it could still meet the requirements of the standard for the pullout force when the equipment is in service. In addition, during grooved expansion, the pullout force drop caused by elastic hysteresis is smaller than it is in the joint without groove [20]; thus, the pullout force value is more conservative. Although lines A and B coincided in Figure 11, the values of the pulling force in these two cases were quite different, indicating that the pullout force is related not only to the residual contact pressure but also to the deformation of the heat exchange tube near the groove.

**Figure 13:**
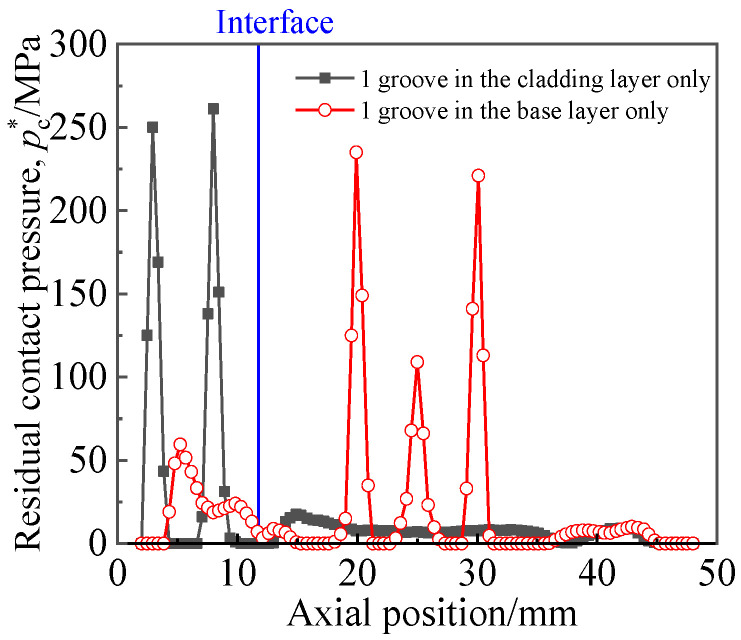
Axial distribution of residual contact pressure.

Figure 13 depicts the distribution of residual contact pressure along the axial path at the position of circumfluence 0° when the expansion pressure was 280 MPa. Only two cases are considered in Figure 13: a single groove in the cladding and a single groove in the base. It can be seen from Figure 13 that, at the corner of each side of the groove, the residual contact pressure was high because deformed tubes sank into the groove during expansion. Both the tube and the cladding layer were titanium materials, and the rebound step was consistent. Therefore, the maximum residual contact pressure between the titanium tube and the titanium cladding layer was greater than that between the titanium tube and the Q345R base layer. The titanium pipe sank more deeply in the cladding groove than in the base groove, and the pullout force was correspondingly greater.

### 4.2. Effect of Groove Width

**Figure 14:**
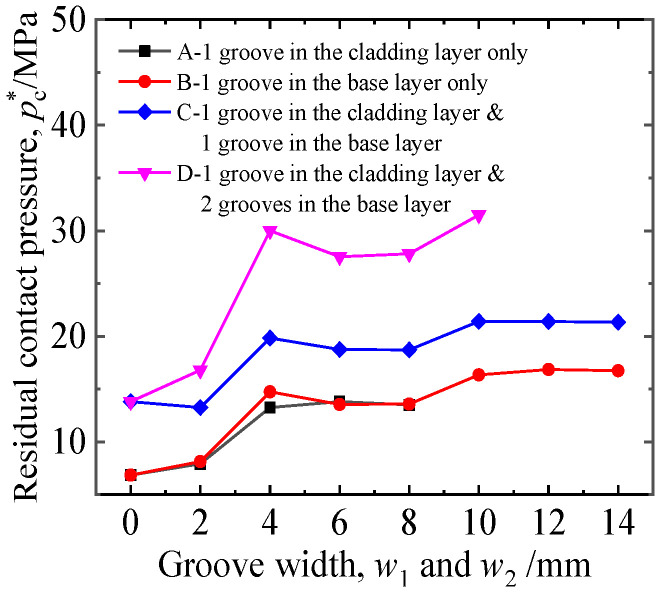
Effects of groove width on residual contact pressure.

Figure 14 shows the influence of groove width on joint performance when the expansion pressure was 280 MPa, the groove depth was 0.5 mm, and four grooving locations were considered. If the expansion length allowed, the groove width was as large as possible. It can be seen from the results in Figure 14 that the groove width had a great influence on *p*_c_^*^. With the increase in groove width, *p*_c_^*^ showed an overall upward trend. When the groove width was 10 mm, *p*_c_^*^ approached the maximum, and the optimal groove width for uniform expansion was generally 8–10 mm [13]. It is worth noting that, when the groove width was 4 mm, *p*_c_^*^ increased significantly and peaked earlier. In this paper, the expansion pressure of 290–320 MPa was also studied. The results show that a larger *p*_c_^*^ was obtained when the groove width was 4 mm.

The reasons were as follows: in the expansion process, the external surface of the tube and the grooved structure always contacted from the edge of the groove, followed by the bottom of the groove, and finally filled the side of the groove. When the groove width was 2 mm, a small part of the titanium tube “sank” into the groove, the concentrated line contact pressure was generated between the tube wall and the groove edge, and the residual contact pressure after unloading increased compared with that without grooving. When the width of the groove increased to 4 mm, the “sinking” effect was greater. However, because the slot width was smaller, the middle of the depression was not in contact with the bottom of the groove, and the titanium tube was close to the edge of the groove. At this time, the *p*_c_^*^ of the outer wall of the tube at the corresponding groove corner increased significantly. When the groove width was 6 mm and 8 mm, the titanium tube and the bottom of the groove contacted, and the corresponding position of the tube outer wall generated residual contact pressure. At the same time, “bottoming” led to a decrease in the titanium tube and groove edge line contact pressure, and the overall effect was a slight decrease in *p*_c_^*^. When the groove width was 10 mm, with the increase in contact area between the titanium tube and the groove bottom, the contact was more sufficient, and the *p*_c_^*^ increased. When the groove width was greater than 10 mm, *p*_c_^*^ showed a slight downward trend.

**Figure 15:**
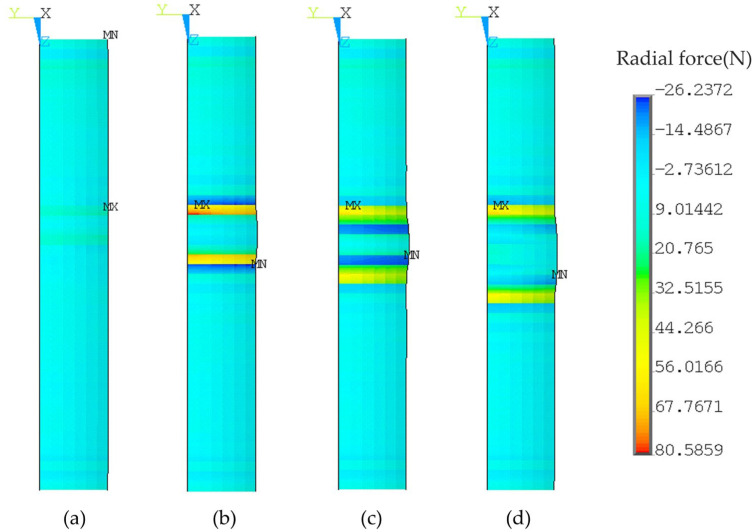
Effects of groove width on radial force at groove edge. (**a**) 2 mm; (**b**) 4 mm; (**c**) 6 mm; (**d**) 8 mm.

The elastic modulus of titanium was small, and the titanium tube could easily “sink” into the groove. Before contact with the bottom of the groove, the titanium tube contacted the edge line of the groove. The groove edge produced a high radial force on the outer surface of the titanium tube, which corresponded to a large *p*_c_^*^. Figure 15 shows the radial force of the outer surface of the titanium tube at the joint with a single groove in the base layer only, and groove widths of 2 mm, 4 mm, 6 mm, and 8 mm. It can be observed that, when the groove width was 4 mm, the radial force was the maximum.

**Figure 16:**
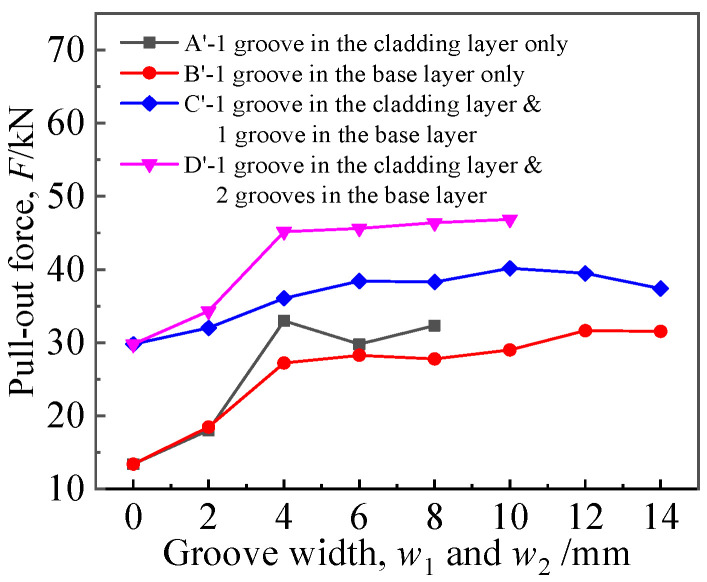
Effects of groove width on pullout force.

As shown in Figure 16, in general, the pullout force increased with the groove width. This is because, with the increase in groove width, the heat exchange tube “sank” into the groove more fully, and the shear force against the tube wall increased; hence, the pullout force increased. However, when the groove width continued to increase, equivalent to the increase in the initial expansion gap, the pullout force declined.

### 4.3. Effects of Groove Location in the Base

**Figure 17:**
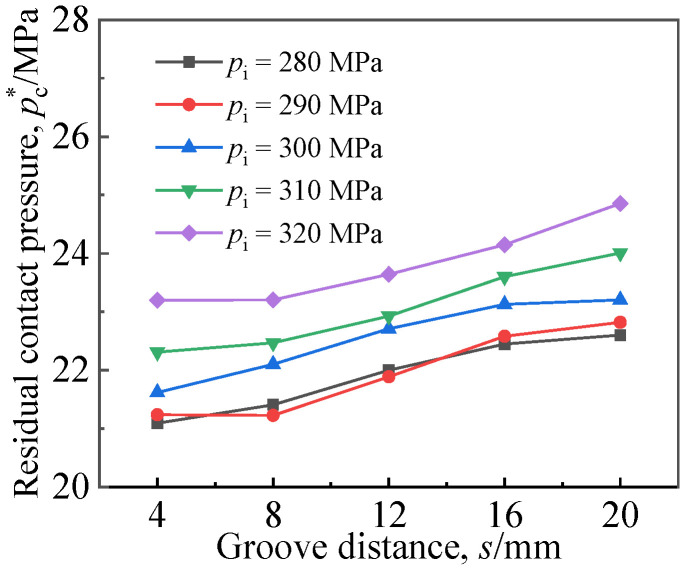
Effects of groove distance from the single groove in base to interface on residual contact pressure of joints.

Figure 17 represents the influence of the distance from the single groove in the base layer to the clad interface on *p*_c_^*^, where *w*_1_ was 6 mm and the width of the single groove in the base layer *w*_2_ was 10 mm. As depicted in Figure 17, with the increase in distance from the single groove in the base layer to the interface, *p*_c_^*^ showed an upward trend. In engineering practice, the distance from the single groove in the base layer to the interface can be appropriately increased to improve the performance of the expanded joint.

**Figure 18:**
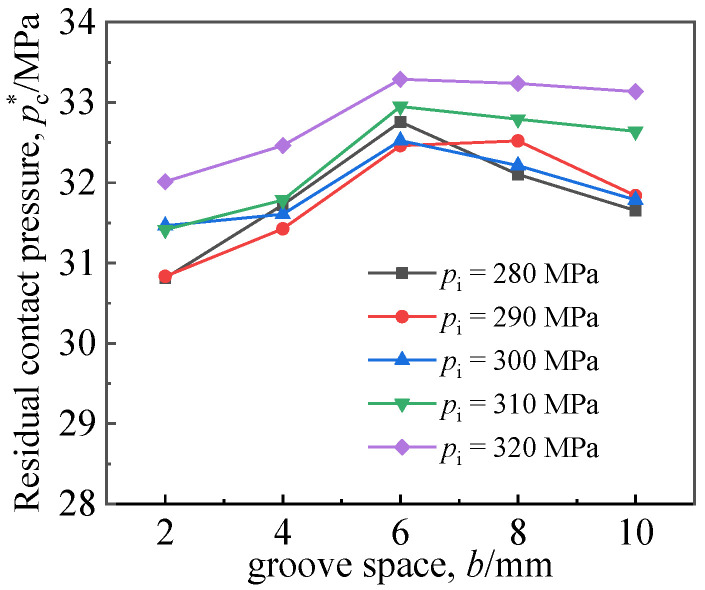
Effects of groove space on residual contact pressure of joints.

Figure 18 shows the influence of double groove space b on *p*_c_^*^. Here, the width of the clad groove *w*_1_ was 6 mm, the width of the double groove in the base layer *w*_2_ was 10 mm, and the distance from the first groove in the base layer to the interface was 4 mm. As can be seen from the figure, the double groove space *b* had little influence on *p*_c_^*^. When the spacing was 6 mm, *p*_c_^*^ exhibited the highest value. 

## 5. Conclusions

In this study, the finite element method was used to explore the influence of different expansion parameters on the performance of the expanded joint between a titanium–steel tubesheet and a titanium tube. The difference between a Q345R tubesheet and TA2-Q345R tubesheet in tight expansion was analyzed, and the effects of groove position and width on residual contact pressure *p*_c_^*^ and pullout force *F* in the TA2-Q345R tubesheet expanded joint were analyzed. The following conclusions could be drawn:

(1) During tight expansion, the clad tubesheet and the tube were both made of titanium, the rebound after the expansion was consistent; thus, *p*_c_^*^ and *F* in the cladding layer were higher than in the base layer. The connection strength of the TA2-Q345R clad tubesheet joint was better than that of the Q345R tubesheet joint.
(2) In the case of single groove, the residual contact pressure of grooving in the cladding layer was equivalent to that in the base layer, while the pullout force of grooving in the cladding layer was higher than that in the base layer. When the number of grooves in the base layer was two, the optimal *p*_c_^*^ and *F* could be obtained.
(3) The expansion performance of the joint was gradually enhanced with the increase in groove width within the range of 2–14 mm. When the groove width was 4 mm, the residual contact pressure increased significantly due to the higher radial force of the groove edge. The position of the groove had no obvious effect on the joint performance in the base layer or in the cladding layer.

## Figures and Tables

**Table 1:** Material properties.

Material	Yield Strength, σ_s_ (MPa)	Elasticity Modulus, E (× 10^5^ MPa)	Poisson’s Ratio, μ
TA2	380	1.1	0.41
Q345R	347	1.95	0.3

**Table 2:** Geometry of the grooves.

Parameter Level	Groove Width *w*_1_ (mm)	Groove Width *w*_2_ (mm)	Groove Distance *S* (mm)	Groove Spacing *B* (mm)
1	2	2	4	2
2	4	4	8	4
3	6	6	12	6
4	8	8	16	8
5		10	20	10
6		12		
7		14		

## Data Availability

Not applicable.
